# Lymphedema after pelvic and para-aortic lymphadenectomy—results of a systematic evaluation in patients with cervical and endometrial carcinoma

**DOI:** 10.1007/s00404-022-06779-8

**Published:** 2022-10-12

**Authors:** R. Armbrust, V. Auletta, G. Cichon, G. Vercellino, K. Yost, J. Sehouli

**Affiliations:** 1 Department of Gynecology with Center for Oncological Surgery, Charité-University Hospital Berlin, Augustenburger Platz 1, 13353 Berlin, Germany; 2 Department of Gynecology, Helios Klinikum Niederberg, Velbert, Germany; 3 Division of Epidemiology, Department of Health Sciences Research, Mayo Clinic, Rochester, USA

**Keywords:** Lymphedema, Lymphadenectomy, Endometrial cancer, Cervical cancer

## Abstract

**Background:**

Lymphedema is a frequent complication after surgical treatment in gynecological oncology with substantial impact on patients´ Quality of Life (QoL). Little is known about screening instruments and prevention. Primary objective was to develop and validate the German version of a 13 items screening questionnaire (SQ) developed by Yost et al. to provide a valid instrument for early diagnosis of lower extremity lymphedema (LEL).

**Methods:**

After translation the SQ was used in pt. with cervical or endometrial cancer who underwent pelvic/paraaortic Lymphadenectomy. Sensitivity and specifity were analysed regarding possible prediction and influencing factors of LEL.

**Results:**

67 pt. had LEL (*N* = 128). Nearly 50% of women in each group (38 in LEL + e 30 in LEL − ) had a body mass index (BMI) > 30 kg/m^2^. Number of removed lymphnodes, radiotherapy and were significantly associated with development of LEL. Translated Mayo Clinic questionnaire can be used with reliable specifity and sensitivity. Four additional questions improved the diagnostic accuracy of the SQ.

**Conclusions:**

The translated SQ is a valuable and predictive tool for screening and early detection of LEL in Gynecological cancer surgery and can even improved by adding simple questions.

## What does this study adds to the clinical work?

**Table:** 

The results of this trial again highlights the Importance of early screening and detection for Lymphedema after gynecological surgery and describes patients at risk better.	

## Introduction

Lymphedema (LE) is a chronic disease of the interstitial compartments and a result of an imbalance in the fluid homeostasis of the lymphatic system [1]. According to the etiology, LE is classified as primary or secondary. Secondary LE develops as a result of a known triggering event, such as infection, radiotherapy or surgical therapy [2].

The actual prevalence of LE is unknown but is likely to be underestimated as patients with latent or mild disease may not receive treatment [3, 4].

In gynecological tumors, the incidence of lymphedema of the lower extremities (LEL) after lymphadenectomy is estimated to be 30–40%; in combination with radiation therapy, the percentage increases to up to 60% in the lower extremity and up to 50% in the upper extremity [5]. However, this also depends on several therapy and patient associated characteristics. Especially patients with cervical or endometrial cancer develop LEL due to the frequency of pelvic and /or paraaortic lymphadenectomy.

The incidence of LE patients with cervical cancer varies between 3.6% and 30.2% [7]. Patients who have undergone secondary lymphadenectomy for endometrial cancer develop LEL in 1.2–37.8% [8]. The risk of lymphedema is related to the number of lymph nodes removed. Some authors have shown that the removal of more than 25 lymph nodes, as well as radiotherapy and the removal of the deep iliac lymph nodes increases the risk of lymphedema of the lower extremities [9, 10]. The latency between surgery and clinical symptoms varies widely. A distinction is made between an early occurrence 6 months after an operation and a late occurrence up to 10 years after an operation [6].

The most important factor in successful treatment of lymphedema is early diagnosis. The main goals are to slow down progression, to interrupt the increase in the circumference of the extremity, to avoid complications and finally to prevent further functional impairment [11, 12].

The Mayo Clinic developed and validated a questionnaire for screening secondary LEL in 2012. As a template for developing the questionnaire, the Mayo Clinic team used a questionnaire that was originally developed for the early diagnosis of LE of the upper extremity (after surgical treatment of breast cancer) [13].

The aim of the present study was to evaluate the diagnostic accuracy of this (translated in German language) questionnaire of patients who underwent pelvic and/or para-aortic lymphadenectomy within the treatment of endometrial or cervical cancer. The primary objective of this trial was to evaluate the questionnaire regarding several different cutoff measurements (as in the American questionnaire of the Mayo Clinic) as well as evaluation of sensitivity and specificity and to better describe patients characteristics with LEL. In addition, the weighting of risk factors was verified with the help of retrospective statistical analyzes. The present results also represent the first analysis of the questionnaire in Germany.

## Materials and methods

The questionnaire consists of a total of 13 questions (items) relating to all subjective symptoms, in particular swelling, pain, discomfort, stiffness, tension and numbness. Four additional questions (items) were added (details can be found in the supplemental material). The answers are recorded using a Likert scale. Depending on the degree of severity, the score ranges from 0 (absent) to 4 (very pronounced). A total number of points is determined by adding up the individual points for each item. In addition, the last question records the extent to which the diagnosis of lymphedema has already been made by medical staff.

Inclusion criteria: patients with cervical and endometrial cancer who were at least 18 years, who did not have pre-existing lymphedema and who had to undergo a pelvic e/o para-aortic lymphadenectomy as part of the surgical treatment of the carcinoma.

A total of 200 patients were contacted. Total of 128 data sets were included in the evaluation (Table. 1). In January 2019, the patient was contacted again by phone to obtain information about the course of the lymphedema.

**Table 1:** Sociodemographical and patients characteristics

	Average	*N*	Histology	*N*	%
Age	58.2 (28–57)	128	Cervical cancer—adenocarcinoma	30	23.4
BMI	27,8 (17.1–50)	128	Cervical cancer—neuroendocrine carcinoma	1	0.8
			Cervical cancer–squamous epithelial	43	33.6
	*N*	%	Endometrium cancer—adenocarcinoma	47	36.8
BMI < 30	68	53.1			
BMI > 30	60	46.9	Tumorstage for Cervicalcancer	*N*	%
			IA	4	5.4
Secondary diagnosis	*N*	%	IA1	2	2.6
Arterial hypertension	8	6.2	IA2	7	9.4
Thrombosis	4	3.1	IB	5	7.2
Lymphocele	2	1.6	IB1	45	60.8
Coagulation problems	1	0.8	IB2	2	2.6
Ovariancancer	1	0.8	IIA	1	1.3
Breastcancer	1	0.8	IIA2	1	1.3
VIN III	1	0.8	IIB	4	5.4
Restless-legs-syndrom	1	0.8	No data	3	4
Cardiac insufficiency	1	0.8			
No secondary diagnosis	102	79.7	Nodalstage for cervicalcancer	*N*	%
No data	6	4,6	N0	66	89.2
			N1	5	6,8
			G2	13	27.7
			G3	5	10.6
			No data	7	14.9

Questionnaires were sent mail between 2006 and 2015 with some interruptions. The patients were contacted also by telephone in 06/2016 and 01/2019 to complete the follow-up.

### Statistical analysis

The present study is a monocentric, observational study. The data collection and the statistical evaluation were carried out by the software program SPSS 20.0 for Windows. Descriptive statistics were used to analyze the data. This includes the following statistical values: mean, median, standard deviation. The frequencies calculated as a percentage have been rounded to the first decimal place. The reliability of the questionnaire and its ability to reproduce the same results for the same situation was calculated using the Cronbach's alpha coefficient. The selected test procedure was carried out using the following criteria: number of comparison groups, form of distribution, homogeneity of variance, scale variables. The statistically significant difference between the medians of the two groups LE + and LE− for the 13-point questionnaire and subsequently for the 17-point questionnaire was checked using the Mann–Whitney *U* test. The predictive validity of the questionnaire and the specificity and sensitivity of the data collected as well as the relationship between various factors and the development of lymphedema were determined using the chi-square test, Fisher’s exact test, the ROC curve and the logistics Model checked. The statistical significance was set at *p* < 0.05 and *p *< 0.001.

## Results

Between January 2007 and December 2014 (84 months), 200 lymphadenectomies by (mainly) laparoscopy and laparotomy were performed in women with cervical and endometrial cancer in the Dept. of Gynecology, Charité University Hospital BerlinCampus Benjamin Franklin, Campus Mitte and Campus Virchow Klinikum). The sociodemographic and clinical characteristics of the patients are summed up in Tables 1 and 2.

**Table 2:** Surgical and treatment characteristics

Hysterectomy	*N*	%
Hysterectomy mit adnexectomy	84	65.6
Hysterectomy whithout adnexectomy	41	32
No data	3	2.4

Typ of hysterectomy	*N*	%
Abdominal hysterectomy	13	10.2
Laparoscopy (TLH/LASH/LAVH)	77	60.2
Radical hysterectomy (Wertheim–Meigs)	35	27.2
Trachelectomy	3	2.4

Typ of Lympadenecotmy	*N*	%
Pelvinen lymphadenectomy	52	40.6
Pelvinen und paraaortal lymphadenectomy	76	59.4

Radiochemotherapy	*N*	%
Brachytherapy	8	6.3
Radiochemotherapy	30	23.4
No therapy	84	65.6
No data	6	4.7

**Table 3:** Results of the multivariate logistic regression analysis

	*P* value	Odds ratio	95% Confidence interval	
			Lower	upper
Age < 50	0.001	6.902	2.244	21.232
Radiotherapy	0.080	2.703	0.888	8.222
No. of removed Nodes > 20	0.054	3.168	0.980	10.235
Group A	0.000 (< 0,001)			
Group B	0.000 (< 0,001)	11.262	2.904	43.678
Group C	0.000 (< 0,001)	24.396	6.851	86.863

At the time of the survey, 50% of the patients (64 of 128 women) had postoperatively lymphedema. In 6 patients (9.3%) the edema was limited to one leg, while in 54 patients (84, 3%) lymphedema occurred in both lower extremities. In four patients (6.2%) an edema of the labia was detectable in addition to the bilateral leg edema. Unfortunately, no precise topical information was given for one patient (1.6%). Due to methodological issues the latency period between the lymphadenectomy and the occurrence of the lymphedema could not be precisely determined (Table 3).

In the follow-up, a total of 84 patients (65.6%) were able to conduct another telephone survey. In six women of this group (18%) the lymphedema had worsened. In the remaining 27 women, the degree of edema remained the same. There was no improvement in lymph drainage in any of the patients.

### Statistical significant risk factors for developing lymphedema: age, number of removed lymphnodes and adjuvant radiochemotherapy

To investigate a possible correlation between the age of the patients and the risk of developing lymphedema, a cutoff of 50 years was defined using the ROC curve. About 64.7% of the patients with lymphedema were younger than 50 years, but 32.2% of the patients with lymphedema were older than 50 years. This difference was assessed as statistically significant according to Fisher’s exact test.

It was also investigated to what extent the number of removed lymph nodes correlated with the risk of developing lymphedema. In each patient with lymphedema, an average of 30.1 (SD 16.7) lymph nodes were removed. In patients without lymphedema significantly less lymphnodes were removed (median 21, p = 0.007). Therefore, a cutoff of 20 lymph nodes was taken to identify the number of lymph nodes removed as a possible risk factor for the development of lymphedema.

In 38 (31.2%) of 122 patients, radiochemotherapy was carried out in addition to surgical therapy. Of this group, 27 patients (71.1%) developed LEL, 11 patients (28.9%) did not. Postoperative radiochemotherapy was highly significant associated with development of LEL (*p* = 0.002).

**Fig. 1:**
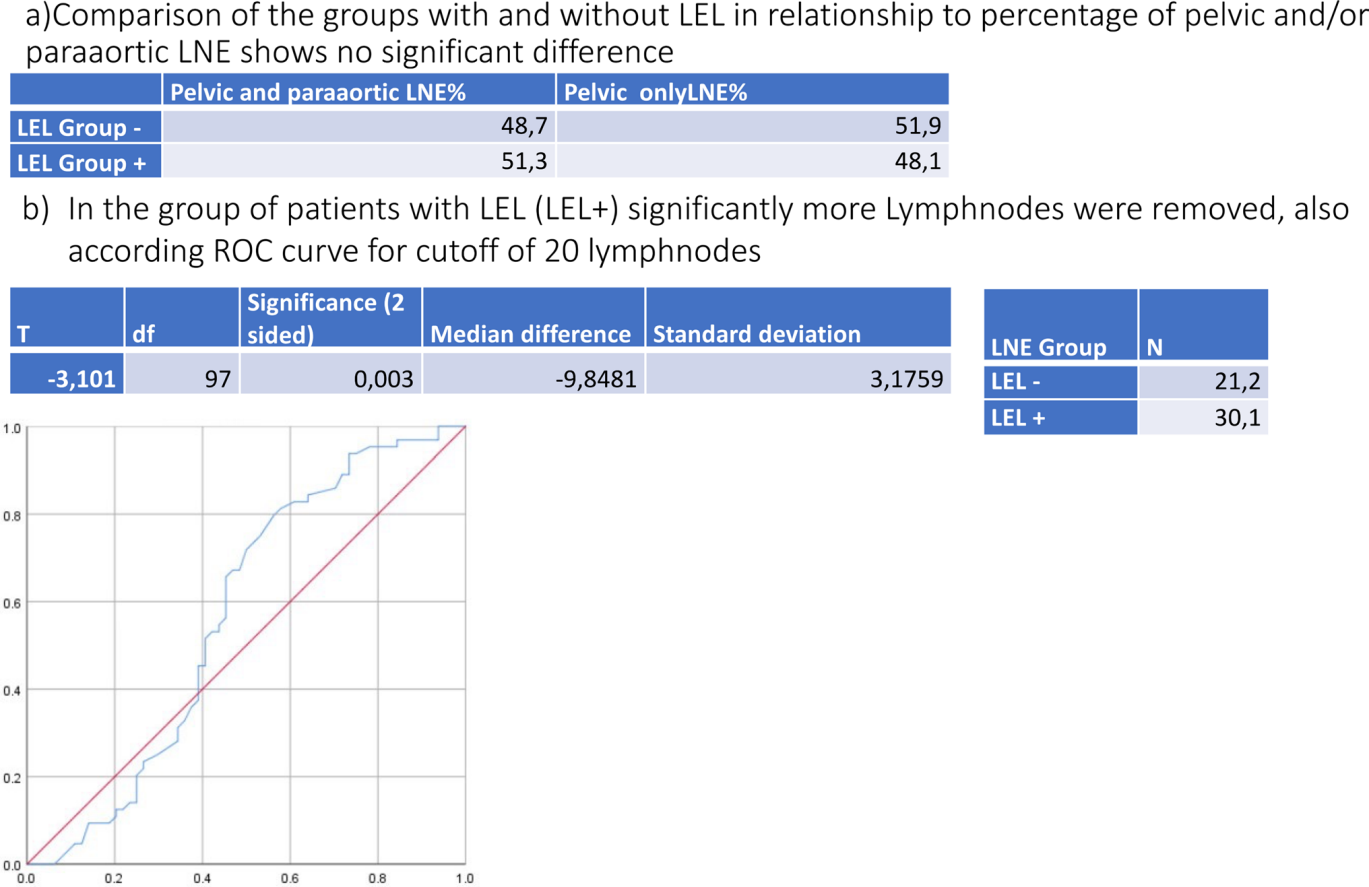
Comparison of groups according to percentage and number of removed lymphnodes

Detailed information can be found in Fig. 1.

#### Exploratory data analysis

The patient collective was divided into two groups. LEL + : patients with lymphedema LEL − : patients without lymphedema. Both groups answered the questionnaire with the same number of points.

**Fig. 2:**
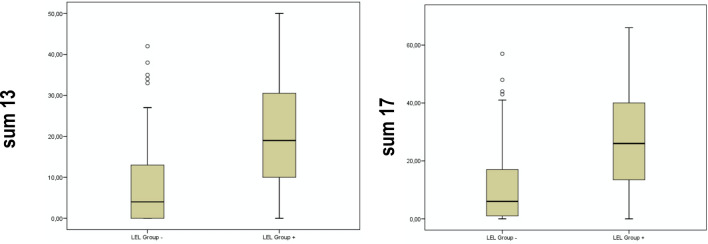
Results of the Mann–Whitney *U* test for the comparison of the model with 13 items vs. 17 items; Cronbach’s alpha = 0.944

The maximum achievable total score for a questionnaire with 13 items is 50. In the group of patients without lymphedema, the mean value of the total score was 4.0 and in the group of patients with lymphedema it was 22.0. The Mann–Whitney *U* test for mean differences between the two groups produced a statistically highly significant result: LEL positive patients had a significantly higher total score for the questionnaire with 13 items. This evaluation was also carried out for the scale with 17 items (*p* < 0.05), Fig. 2.

#### Analysis of the reliability for the 13 and 17 suggested items

Reliability represents the accuracy and replicability with which the questionnaire can detect lymphedema. The reliability (internal consistency) of the 13 items is determined using the Cronbach *α* method—the value of *α* = 0.944 indicates a very high measurement accuracy and thus the reliability for the 13 items.

In a second analysis, the reliability for the 17 suggested items are also examined. The value of *α* = 0.959 also shows a very high reliability for the 17 items.

### Analysis of the specificity and sensitivity for the questionnaire with 13 items with a cutoff value of 4

The calculations resulted in a diagnostic specificity of 36/64 = 56.2% and a diagnostic sensitivity of 59/64 = 92.2% in the entire patient collective for the questionnaire with 13 items. It showed a diagnostic specificity of 23/33 = 69.7% and a diagnostic sensitivity of 32/35 = 91.4% in the patient collective with a BMI < 30 kg/m^2^. In comparison, there was a diagnostic specificity of 13/31 = 41.9% and a diagnostic sensitivity of 27/29 = 93.1% in the patient collective with a BMI > 30 kg/m^2^.

### Analysis of the specificity and sensitivity for the questionnaire with 13 items in patients with a BMI < 30 kg/m^2^ and BMI > 30 kg/m^2^

It showed a diagnostic specificity of 23/33 = 69.7% and a diagnostic sensitivity of 32/35 = 91.4% in the patient collective with a BMI < 30 kg/m^2^. The result was a diagnostic specificity of 13/31 = 41.9% and a diagnostic sensitivity of 27/29 = 93.1% in the patient collective with a BMI > 30 kg/m^2^.

### Analysis of the specificity and sensitivity for the questionnaire with 13

#### Items for patients with BMI < 30 kg/m^2^ and with a BMI > 30 kg/m^2^ according to the ROC curve

The calculations here result in a diagnostic specificity of 84.8% and a diagnostic sensitivity of 85.7% with a cutoff value of 9 in the patient collective with a BMI < 30 kg/m^2^. The diagnostic specificity is 93.9% and the diagnostic sensitivity 62.9% with a cutoff value of 14 in the group of patients with a BMI < 30 kg/m^2^.

A significant difference was found between the two groups (Kendall’s tau b: − 0.44, *p* < 0.001). The calculations resulted in a diagnostic specificity of 54.8% and a diagnostic sensitivity of 86.2% with a cutoff value of 11 in the group of patients with a BMI > 30 kg/m^2^. The diagnostic specificity is 77.4% and the diagnostic sensitivity 62.1% with a cutoff value of 18 in the group of patients with a BMI > 30 kg/m^2^.

### Multivariate logistic regression for the group of patients with lymphedema

Furthermore, multivariate regression analysis was performed: the univariate analysis shows that in our collective the variables BMI, histology of the carcinoma and stages of working according to FIGO have no influence on the development of lymphedema.

The variable with the strongest predictive power is a number of more than 20 lymph nodes removed. The probability of developing lymphedema increases by almost five times (in the CI from 1.72 to 11.47) in the patient collective, where more than 20 lymph nodes were removed. Furthermore, an age of less than 50 years and additional radiotherapy play a decisive role. Based on these results, the influence of these different variables on each other and in relation to the questionnaire was examined. Then, our extended question was: to what extent does the probability of having lymphedema increase in the group of patients who responded with the highest number of points, taking into account the other decisive variables. Since the patients with different BMI also had different cutoff values, a new table had to be created in which these differences are taken into account. Three groups were formed. Group a: a cutoff value < 8 points was used for patients with a BMI < 30 kg/m^2^. A cutoff value < 12 points was used for patients with a BMI > 30 kg/m^2^. Group b: a cutoff value between 8 and 14 points was used for patients with a BMI < 30 kg/m^2^, but a cutoff value between 11 and 18 points was set for patients with a BMI > 30 kg/m^2^. Group c: a cutoff > 14 points was used for patients with a BMI < 30 kg/m^2^, while a cutoff > 18 was used for patients with a BMI > 30 kg/m^2^. In the following it was compared whether the percentage values of group a and group c differ significantly from one another. A significant difference was found between the two groups.

The static significant variable with the strongest predictive power is group c. The probability of having lymphedema increases by almost 25-fold in the group of the patient collective who answered with the most points (in the CI from 1.72 to 11.47).

## Discussion

For many patients with gynecological cancer, secondary lymphedema of the lower extremities is one of the most debilitating secondary effects of surgical and/or radiotherapeutic treatment. When the symptoms and local effects of lymphedema are present, they can only be alleviated, not cured. The resulting disability can lead to severe lifelong morbidity [11, 12].

While there are several studies on the risk factors that lead to lymphedema after axillary dissection in breast cancer, the data on lymphedema after pelvic and para-aortic lymphadenectomy are very limited.

Early signs and symptoms of lower extremity lymphedema are often not recognized or ignored. Diagnosis can be difficult, because the disease often occurs bilaterally, making comparison with an unaffected contralateral extremity impossible (in contrast to upper extremity lymphedema in axillary lymphadenectomy in breast cancer) [14, 15].

In this study an attempt was made to develop a tool (questionnaire) in German that would facilitate the detection of early symptoms for the development of lymphedema of the lower extremities.

For this purpose, a questionnaire developed and validated for the first time at the Mayo Clinic was translated into German [13]. The aim of the present work was to use this questionnaire to record symptoms of lymphedema and to quantify their severity in a point system. With the presentation of the symptoms that are present in a pronounced form in the case of manifest lymphedema and that are already present in the early development phase of lymphedema in a weakened form and can be queried, an attempt was made to determine a threshold value (score) which, when reached, would already result in early forms of lymphedema could be clinically detectable with a high probability or their timely occurrence is likely.

The target group of the survey were patients who had to undergo surgical therapy with pelvic or pelvic and para-aortic lymphadenectomy due to cervical or endometrial cancer. So far, there is no correspondingly validated questionnaire in German-speaking countries. To check to what extent the internal consistency of the questions (reliability) has decreased as a result of the translation into German, a reliability test according to Cronbach was carried out on the basis of the answers given, which showed a Cronbach's α value of 0.944, which is a high internal consistency also speaks of the German questionnaire. In the American study, a sensitivity of 95% and a specificity of 86.5% were achieved from a point value (cutoff value) of 4 [13]. Based on the same cutoff value, the sensitivity of the German questionnaire was slightly flatter at 92.2% and the specificity at 56.2% was significantly lower. The reasons for the divergence with regard to the specificities between the German and the American questionnaire may be due to the different inclusion criteria of the two studies. In the American study, the control group consisted of patients with known lymphedema of the arms, i.e., a collective who had this disease before entering the study. Since these patients are better able to describe symptoms, such as changes in the skin or swelling of the adipose tissue, they influence the specificity. In the present study, however, the control group also consists of women without lymphedema, so that the probability of false negatives increases.

It was examined what influence an increase in the cutoff value has on the specificity of the questionnaire without significantly reducing the sensitivity. To this end, 3 groups were formed:

- cutoff < 9
- cutoff between 9 and 18
- cutoff > 18

For each of the three groups, specificity and sensitivity were calculated taking the BMI into account. For the group with the cutoff value of 9, the specificity is 64.1% and the sensitivity of the questionnaire is 85.9%. With the cutoff value of 18, the specificity increases to 87.5%, while the sensitivity drops significantly at 57.8%. An increase in the cutoff value leads to a higher specificity but at the price of a decrease in sensitivity.

The specificity of the results was not significantly changed by considering four additional questions. The sensitivity of the 17 item questionnaire, on the other hand, was 93.7% for patients in the entire cohort.

If the influence of body weight is taken into account and the collective is divided into normal weight and obese women (BMI > 30 kg/m^2^), the specificity in the German study with a cutoff value of 4 in obese women decreases further to 41.9%, while in the corresponding American collective remains relatively high at 76.5%.

In obese patients, the specificity was lower for both the English and the German questionnaires. This relationship was also found in breast cancer [15]. The assessment of the edema on the lower and upper extremities is missing in the patients with a BMI > 30 kg/m^2^ heavier due to the adipose tissue. This factor can raise the group of false negatives.

For this reason, the sensitivity and specificity were analyzed in two different groups of patients with BMI < 30 kg/m^2^ and BMI > 30 kg/m^2^ with a high cutoff value. In the group of patients with a BMI > 30 kg /m^2^ with a cutoff value of 11, the sensitivity is 86.2% and the specificity 54.8%.

In the collective of patients with a BMI < 30 kg/m^2^ with a cutoff value of 9, the sensitivity was 85.7% and the specificity 84.8%.

In summary, it can be stated that the questionnaire can be used for obese patients as well as for patients of normal weight based on a cutoff value. However, the use of a cutoff value of 9 in patients with normal weight leads to a further increase in the specificity without the sensitivity dropping significantly. The studies show that a questionnaire can become an unencumbered and inexpensive instrument for the screening of lymphedema. These data support the finding that the lymphedema symptoms assessed by patients are more sensitive than the clinical examination [16]. In addition to investigations into the predictive value of a questionnaire for the early detection of postoperative lymphedema, an attempt was made to identify additional risk factors for the development of lymphedema when evaluating the patient data. The evaluations show that about half of the patients develop lymphedema of the lower extremities as a result of a previous lymphadenectomy for cervical and/or endometrial cancer. It was unclear which additional risk and influencing factors seem to favor the occurrence of postoperative lymphedema in our study group. For this reason, the number of lymph nodes removed, the age of the patients, the BMI, venous insufficiency and hypertension were considered as possible influencing and risk factors.

In our study, the number of the extirpated lymph nodes was identified as an independent risk factor for the occurrence of lymphedema. The number of lymph nodes removed as part of the lymphadenectomy was between 8 and 57, with an average of 28. From a number of 20 lymph nodes removed, the probability of developing lymphedema increased to 58.4% (CI 95%). A positive correlation between the number of lymph nodes removed and the edema risk is also seen by Kim et al. [17] and Mitra et al. [8]. However, there are slight differences between the studies regarding the number of removed lymph nodes from which the risk increases significantly. While Mitra et al. [8] already see a significantly increased risk of edema from a number of 10 lymph nodes (odds ratio 5.6, CI 95%), Kim et al. [17] with 30 removed lymph nodes (odds ratio 3.2, CI 95%) significantly higher. In addition, no statistically significant difference between a purely pelvic and a combined pelvic and para-aortic lymphadenectomy was found in our study. In our study there was no statistically significant difference with regard to the influence of obesity. This result contrasts with the results of the study by Kuroda et al. [16] and also Yost et al. [13] who both demonstrate a positive relationship between BMI and lymphedema. In our study, no significant association was found between arterial hypertension and venous insufficiency. These results could be related to the low number of patients with secondary diagnoses. Furthermore, postoperative radiotherapy could be identified as an independent risk factor. This finding agrees with the results of Mitra et al. [8] and was to be expected. The therapeutic effect of radiotherapy is based on the fibrosis of tumor-associated vessels, which ultimately leads to an insufficient supply of the tumor.

However, this effect is bought at the price of the fibrosis of lymphatic vessels, which results in disorders of lymphatic drainage and an increased risk of edema. Unexpectedly in our study, there was a connection between the age of the patients and the risk of edema, which, however, showed an increased risk of edema in younger patients. If one assumes that the contractility of lymph vessels and veins decreases with increasing age, this correlation does not appear to be easily explicable. It may have been an unusual random distribution or a hidden bias.

### Multi-factorial consideration

The extent to which there is a multifactorial relationship between the risk factors age, chemoradiation, number of removed lymph nodes > 20 and group c of the questionnaire for evaluating the existence of lymphedema after pelvic and/or para-aortic lymphadenectomy was also examined. The results of our work show that in a multivariate logistic regression an age younger than 50 years has an OR of 6.9. A number of > 20 removed lymph nodes has an OR of 3.1 for a confidence interval of 95%. The result is that patients under 50 years of age are approximately 6 times more likely to have lymphedema. Patients with more than 18 points in the questionnaire have an approximately 24-fold higher risk of lymphedema (OR 24.3).

### Limitations of the study

The investigation was a retrospective study in which only patients were included who received external follow-up care. The diagnosis of lymphedema was, therefore, made as part of the outpatient follow-up care and communicated in writing and by telephone and not verified in person in a timely manner. This approach harbors the risk of cognitive bias (so-called re-call bias) in which patients can no longer remember exactly the symptoms afterward or interpret and reinterpret them differently in the light of the research question.

With regard to the point in time at which the lymphedema developed and the timing of therapeutic measures, the information in the questionnaires was in some cases not always complete. To be able to further validate the results obtained here and to be able to further increase the specificity of the questionnaire used, a systematic and time-coordinated survey would make sense in addition to a higher number of cases. In addition, it would make sense to carry out future surveys in a prospectively standardized and multicenter framework.

Recent guidelines recommend introducing the Sentinal Node Concept for patients with early stage cervical cancer and endometrial cancer.

## Conclusions

In the early stages, patients with cervical or endometrial cancer often have a very good prognosis. Regardless of the oncological result, long-term effects of therapy such as lymphedema can have a long-term, severely impairing influence on quality of life. When choosing operative and adjuvant therapy (especially radiation therapy), the prognostic gain and side effects must be carefully weighed against each other to avoid potentially serious over-treatment. An important goal of the pre- and post-treatment should, therefore, not only be the timely detection of lymphedema, but above all the early identification of high-risk patients. In both cases, the early and timely initiation of therapy is of decisive importance. Despite the mentioned limitations of the study, it could be shown that the questionnaire presented here can be a convenient, simple and fast aid for doctors for the early detection of lymphedema of the lower extremity.

The handing out of a questionnaire for the detection of early symptoms of lymphedema directly upon discharge from surgical treatment or as part of the first follow-up examination is, therefore, a measure that can help to identify symptoms of the onset of lymphedema at an early stage. In this way, rehabilitation measures could be started at an early stage, which on one hand can have a positive influence on the further course of the suffering and the quality of life of the patients and on the other hand can help relieve the health system of serious follow-up costs.

## Patents

This section is not mandatory but may be added if there are patents resulting from the work reported in this manuscript.
